# Microbial Primer: Mirror bacteria

**DOI:** 10.1099/mic.0.001784

**Published:** 2026-10-09

**Authors:** Vaughn S. Cooper, Jolien M. Sweere

**Affiliations:** 1 Microbiology and Molecular Genetics, University of Pittsburgh School of Medicine, 450 Technology Drive, Pittsburgh, PA, USA; 2 Mirror Biology Dialogues Fund, Dover, Delaware, USA

**Keywords:** biosecurity, chirality, synthetic biology

## Abstract

Mirror bacteria are hypothetical synthetic organisms built from molecules of opposite chirality to those used throughout nature. This primer discusses their potential dangers: because of their chiral mismatch to the natural environment, mirror bacteria could plausibly evade predation, immunity and other ecological control mechanisms to a degree never seen before. This property could simultaneously make them unprecedented invasive species and dangerous pathogens.

## Introduction

The atoms in many molecules can be arranged into one of two stereoisomers – non-superimposable mirror images of each other, labelled d or l by convention – a property known as chirality. Biomolecules are typically chiral: all nucleic acids, most sugars, many lipids and all amino acids except glycine possess this handedness. Remarkably, all life on Earth shares the same handedness: DNA and RNA use d-ribose across all species, never l-ribose; proteins synthesized by the ribosome are built from l-amino acids, although d-amino acids can naturally be incorporated into bacterial peptidoglycan and non-ribosomal peptides.

Why and how this conserved chirality (also called homochirality) arose remains a compelling mystery. A common explanation involves a process of ‘symmetry-breaking’ that led to a bias in favour of d- or l-form molecules that was followed by a process of amplification that reinforced that bias. Regardless of the processes that led to Earth’s biological homochirality, natural-chirality molecules do not appear to be inherently superior to their mirror images. Mirror-image DNA from mirror-image nucleic acids would store genetic information just as effectively as natural-chirality DNA (although its helix would turn in the opposite direction). Likewise, mirror-image enzymes, composed of mirror-image amino acids, would catalyse the same reactions – just mirrored (Fig. 1).

**Fig. 1.**
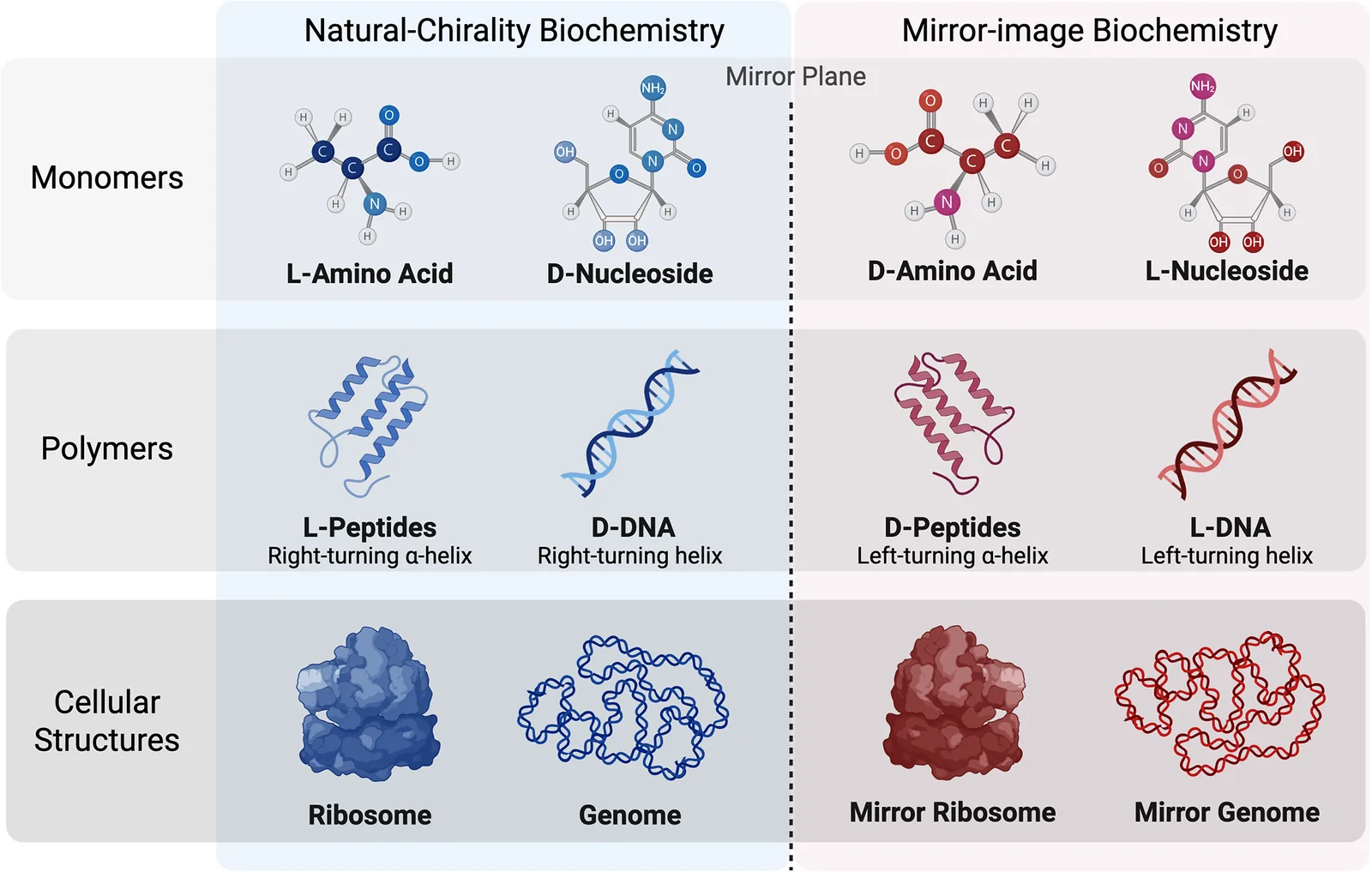
Peptides composed of chiral amino acids should fold into equivalent yet opposite mirror proteins, and nucleic acids form into opposite-turning helices.

This concept extends to entire mirrored self-replicating organisms whose molecular machinery is the mirror image of a natural organism. The simplest such living mirrored organism would be a mirror bacterium. Mirror bacteria cannot emerge naturally from a natural-chirality world, as nature would have to re-evolve life from scratch, but creating them artificially in the lab increasingly appears to be technologically feasible. Yet the asymmetries in their expected interactions with natural-chirality life could plausibly pose extreme and unprecedented dangers to humans and the environment. In this primer, we will discuss both of these issues.

## How could mirror bacteria be made?

Several pathways towards mirror bacteria have been proposed. One prominent approach is bottom-up: producing mirror images of the key cellular components (e.g. DNA, lipid membranes and ribosomes) and assembling them into a functional cell. The second, top-down approach, involves grafting mirror-image biochemistry into a natural-chirality organism piece by piece, slowly converting it into a mirror-image bacterium.

Both are much easier said than done. Mirror building blocks, like amino acids and nucleic acids, are straightforward to synthesize chemically, but combining them into mirror macromolecules is much harder. Producing a mirror RNA polymerase was a recent breakthrough, but cellular organelles, e.g. mirror ribosomes, remain a much greater challenge. And even if all the ingredients can be produced, assembling them into a living organism has never been achieved in either chirality.

This makes timelines hard to predict. Breakthroughs can turn major roadblocks into minor ones; steps that look simple can prove surprisingly stubborn. But significant advances in chemistry have accelerated peptide synthesis in both d- and l-forms, and kilobase-length nucleic acids are now routine and can be produced in the l-form. These advances in chemical synthesis, combined with diverse new efforts to build synthetic cell components and cell booting approaches, suggest that mirror bacteria could be plausibly created within the next few decades.

Mirror bacteria do not yet exist, so no one has been able to study them directly. This might seem to undermine confidence in the risk assessment that follows. Yet the long-range picture is unusually clear for a hypothetical technology. Mirror molecules have been synthesized and studied for decades, and their interactions with various natural-chirality biological systems have been characterized. The stereospecificity of molecular interactions can all be examined using existing mirror-image compounds without a mirror bacterium in hand. Models and natural analogues, such as existing immunodeficiencies, invasive species dynamics and known bacterial pathogens, provide further evidence of the form and scale of the potential risks described below.

## Mirror bacteria in the wild

The first mirror microbes would likely be fragile and survive only in strictly defined lab conditions, which would serve as a form of containment. However, these limitations would be relatively straightforward to overcome: adding pathways required for improved metabolism or stress tolerance is far simpler than creating self-replicating cells in the first place. In principle, a wide range of bacterial species could eventually be mirrored, with the likely start being the common model strain *Escherichia coli*. Other model heterotrophic laboratory species like *Bacillus subtilis* are possibilities but also phototrophic species such as *Prochlorococcus*. Given the vast available knowledge and resources for *E. coli*, we use this as our conceptual model.

In laboratory culture alone, mirror *E. coli* would look unremarkable, growing under the same abiotic constraints (temperature, pH and osmolarity) as its natural-chirality counterpart. It is noteworthy how versatile *E. coli* is, with strains growing between 8 and 48 °C and between pH 4 and 9. But its nutritional requirements will be the exact opposite, preferring l-sugars and d-amino acids to the same extent that ordinary cells prefer the reverse. A surprising number of d-amino acids can be utilized by *E. coli,* however, suggesting the mirror version could access their l-version equivalents. The nitrogen, phosphorus and sulphur that bacteria need are accessible as achiral ions such as ammonium, phosphate and sulphate, which a mirror bacterium would be expected to take up as readily as any other, leaving organic carbon as the main potentially limiting nutrient. The relative rarity of opposite-chirality carbon nutrients like l-sugars in nature would likely limit growth of mirror *E. coli*, but many central metabolites like pyruvate and succinate, sugars like glycerol and diverse fatty acids are achiral and have been shown to support *E. coli*’s growth as sole carbon sources. *E. coli* can sustain its growth on individual suitable carbon sources at concentrations as low as 20 µg l^−1^, and achiral carbon nutrients are present in various environments like soil, freshwater and host tissues at sufficiently high concentrations (see for more details). Losing access to abundant typical chiral sources would very likely slow mirror bacterial growth but not starve the population.

Moreover, the diet breadth of mirror bacteria would likely broaden over time as the population expands, acquires mutations at rates comparable to ordinary *E. coli* and experiences natural selection for faster-growing mutants. Some bacterial species can already grow on l-glucose as a sole carbon source, despite its rarity in nature, through off-target enzymatic activity. *E. coli* has also been experimentally evolved to utilize mirror amino acids as carbon sources. Therefore, there are likely a variety of ways by which mirror bacteria could plausibly evolve to overcome their initial nutritional constraints.

We still do not know if the complexity of natural environments containing dozens of substrates that would be foreign to mirror organisms could be directly toxic, and this is deserving of further study. Limited research on effects of mirror-image molecules on natural-chirality bacteria suggests that toxicity from opposite-chirality molecules only occurs at high concentrations not found in nature. Regardless, nutritional restriction would very likely reduce growth rates and disadvantage mirror bacteria relative to natural-chirality competitors.

Once mirror bacteria interact with other organisms, however, competitive advantages switch drastically. Because all life on Earth has evolved under homochirality, all molecular interactions among organisms are engrained with these conformations. Most interactions between species come down to stereoselective receptor–ligand binding, and a mirror bacterium would be chirally mismatched to every organism it encountered. This would grant mirror bacteria unprecedented protection from predators, parasites and bacterial competitors. They would be effectively immune to bacteriophages – the most common cause of bacterial death – since even if a phage could bind to the cell surface, its genetic material would be unreadable. Protists and other grazers would likely have impaired chemotaxis towards reverse-chirality metabolites and could not enzymatically digest the mirror macromolecules they engulf. Many antibacterial weapons that natural-chirality bacteria deploy against each other rely on receptor binding and would likely be ineffective against mirror counterparts.

Population growth requires that births outpace deaths, which are impacted by more than predation. Whether mirror bacteria achieve positive net growth in a given environment depends on whether the limited, mostly achiral nutrient supply sustains division rates above remaining sources of mortality like abiotic causes and competition from other microbes, which are not greatly influenced by chirality. Where that condition is met, mirror lineages could experience ecological release: resistant to the predators that normally constrain *E. coli*, they could spread into diverse niches despite nutritional and abiotic limits.

Natural-chirality organisms could in principle develop equivalent adaptations to a growing mirrored biomass, but the odds are less favourable. Although catabolism of mirror substrates by protists seems evolutionarily feasible, mirror bacteriophages capable of infecting mirror bacteria are highly unlikely to arise through natural selection. More fundamentally, the selective pressures are asymmetric: mirror bacteria would be surrounded by chirally mismatched nutrients from day 1, and any variant more capable of using them would be strongly beneficial, whereas native organisms would only encounter chirally mismatched prey if mirror bacteria were already abundant, limiting selection for usage of mirror nutrients.

Beyond these general adaptations, we could expect mirror bacterial subpopulations to undergo adaptive radiation as they colonize new niches, in essence founding a new branch of life. Chiral mismatch between nucleic acids in the mirror cell and in the environment would prevent recombination with exogenous DNA, but homologous recombination among diversifying lineages could still occur and facilitate exchange of adaptations. While establishing a population in a new environment is a challenge, it is one mirror bacteria could overcome often enough to matter. It has been shown that an l-DNA barcode can be amplified from pond water a year later in comparison to a d-DNA barcode lasting only a day, demonstrating mirror molecules are very resistant to enzymatic degradation. While a full mirror cell would likely also enjoy similar protection against degrading enzymes, cell survival is also dependent on tolerance to abiotic factors, like drying and temperature, which chirality barely affects. The geographic spread of mirror bacteria (via animals, human travel and ocean currents) could be just as fast as other native bacteria. Many arrivals might fail, but any that takes hold would likely be near-impossible to remove, causing risk to accumulate with each introduction. Over time, this could seed invasive lineages across suitable ecosystems worldwide, which could displace members of natural microbiomes that are essential for nutrient cycling. The ecological consequences of such displacement could be severe.

## Infection and immunoevasion

Alongside being a peerless invasive species in the natural environment, mirror bacteria could prove dangerous opportunistic pathogens. The same chiral mismatch that would shield them from predators and competitors in the environment would also make them hard to detect by mammalian – and for that matter, metazoan – immune systems (Fig. 2).

**Fig. 2.**
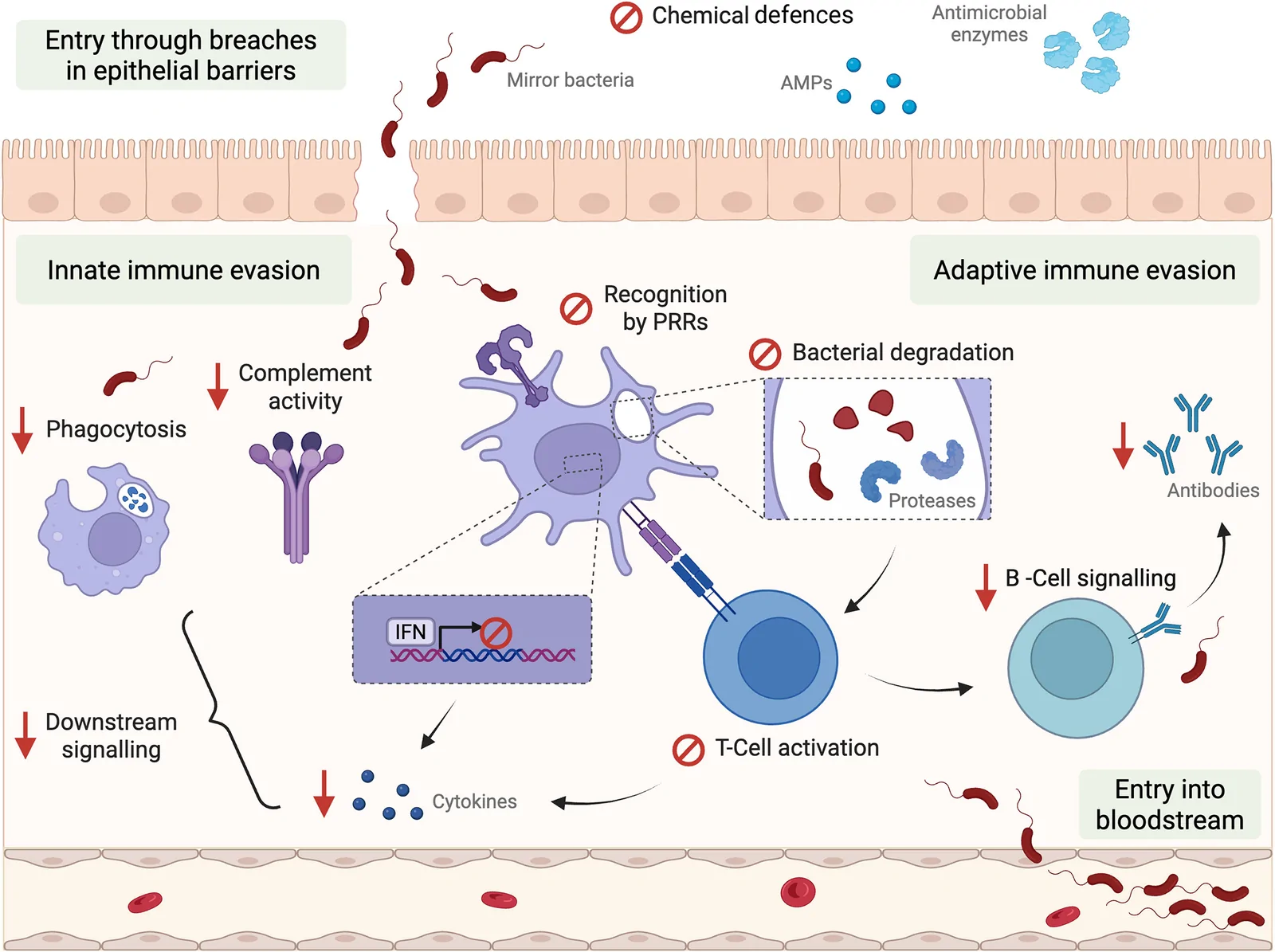
Mirror bacteria would evade multiple layers of mammalian immunity. AMP, associated molecular pattern; PRR, pattern recognition receptor

The innate immune system detects pathogens by binding pathogen-associated molecular patterns (PAMPs) to dedicated pattern recognition receptors (PRRs). Virtually all known PAMPs are chiral, and mirror bacterial PAMPs are unlikely to trigger human PRRs. The effectors of the innate immune system are similarly stereospecific: phagocytosis by neutrophils and macrophages depends on receptor–ligand interactions for chemotaxis, engulfment and digestion, all of which would be heavily impaired against mirror bacteria. Various antimicrobial peptides and complement system activation arms rely on achiral mechanisms and could retain some activity; but how their production would get activated in the absence of PRR-mediated inflammation is unclear.

The adaptive immune system is much more flexible and should in principle be capable of binding mirror bacterial antigens. Indeed, if the adaptive immune system could function normally with this flexibility, mirror bacteria may not be able to infect their hosts. Unfortunately, an effective adaptive response requires more than receptor binding: it also depends on antigen presentation and activation signals from the innate immune system. Since mirror bacteria would largely avoid triggering innate immunity, those activation signals may never arrive. And even if they did, a critical issue is that antigen presentation itself involves a series of stereospecific steps – proteolysis of ingested bacterial proteins, loading of peptides onto MHC-II molecules, antigen presentation to T-cell receptors – each of which would likely be attenuated or non-functional for mirror bacterial antigens.

Immune systems are complex and include mechanisms that are not stereospecific. Some PRRs can bind enantiomers of their ligands, albeit with much lower affinity, and one (uncommon) achiral PAMP has been discovered. Parts of the complement system do not depend on chiral activation mechanisms. Macrophages can internalize latex beads and asbestos without specific receptors, and mirror bacteria would remain vulnerable to reactive oxygen species in the phagolysosome. Some T- and B-cell subtypes are activated independently of classical antigen presentation and may still respond to mirror-bacterial lipids or repetitive molecular motifs. However, these are exceptions within the vast immune repertoire. The general rule is that immunobiochemistry is stereoselective, and the chiral mismatch would leave the human immune system broadly impaired.

It is difficult to model the spectrum of immunodeficiencies resulting from chiral antigenic mismatch because of uncertain and complex interactions among systems. If the functional and impaired arms of the immune system work in parallel, the former could partially compensate for the latter. If they work in series, a single stereoselective weak link could knock out an entire pathway. Unfortunately, effects of single human immunodeficiencies are not reassuring. For example, MyD88/IRAK4 deficiencies effectively knock out a wide range of pattern recognition receptors (i.e. Toll-like receptors fail to elicit inflammatory responses). MHC-II deficiency ablates antigen presentation and most antibody responses. Both disorders cause severe infections from early life, with mortality of 40% or greater despite aggressive treatment. Chiral mismatch would likely impair a broader range of mechanisms than either genetic deficiency. These are not perfect analogies, but they give a sense of what mirror bacterial infection might look like.

Comparing mirror bacteria to known bacterial pathogens is also instructive. Natural-chirality pathogens can mask individual PAMPs, escape phagolysosomes and inactivate antimicrobial proteins – but they typically deploy these strategies one at a time, under strong selective pressure. Chiral mismatch would avoid several detection layers simultaneously. Mirror bacteria would likely be more immunoevasive than any known organism.

It is important to note that the specialized adaptations natural pathogens use to adhere, invade and damage host tissue would likely fail in mirror form. But entry does not require it: breaches in epithelial barriers are common, and once inside, a mirror bacterium faces a similar balance between births and deaths as in the wild. Hosts supply achiral nutrients, richly in the gut lumen and more sparingly in blood and other tissues, so growth could be possible even if slow, provided the mirror bacterium withstands the few host defences that do not depend on chirality. Slowed growth against greatly reduced removal could still add up to a sustained, spreading infection. A mirror bacterium could be dangerous without any of the toxins or invasion machinery that ordinary pathogens rely on, simply by growing at a sustained rate and reaching densities where their sheer presence could cause harm to the host.

The issues described above would extend to animals and plants, as they also rely on chiral molecular patterns to recognize pathogens. While it is difficult to anticipate the diversity of growth-restricting conditions that mirror bacteria may encounter across multicellular hosts, it is quite likely that stereoincompatibility will limit anti-pathogen mechanisms across the tree of natural-chirality life. Absent effective immune clearance, each host becomes a temperate, nutrient-dense environment with sufficient achiral carbon and other nutrient sources for growth. Therefore, even a bacterium lacking conventional virulence factors like bacterial toxins would be extremely dangerous. Mirror bacteria could be devastating accidental pathogens of many natural-chirality species they encounter.

## Containment and countermeasures

The potential danger of mirror bacteria could, in theory, be reduced by strictly preventing their escape into the environment or by developing potent countermeasures to mitigate the consequences if they do escape. Based on current evidence, neither pathway is sufficiently capable of bringing the risks of creating mirror bacteria to acceptable levels.

High-security laboratories can house dangerous microbes, and their sophisticated and layered technical containment methods greatly limit exposure to people and the wider environment. But no containment is perfect. Pathogens can escape such facilities in broadly two ways: through accidental exposure or through deliberate release by a reckless or ill-intentioned actor, and we will consider both scenarios separately.

Even in the most secure facilities, accidents happen: one study recorded that the majority of the roughly 70 cases of human-caused pathogen exposure involving high-risk human pathogens worldwide between 1975 and 2016 were the result of an accident. Limitations in detection and reporting suggest the true incidence is likely higher, although the exact frequency of pathogen escape is uncertain. For an ordinary pathogen, a rare escape is a manageable risk. For mirror bacteria, it may not be, because a single escape that established could plausibly not be reversed. Therefore, containment need not fail often to fail catastrophically.

To limit the risks of accidental release, researchers use synthetic auxotrophy, or engineering bacterial metabolism to depend on artificial nutrients that do not exist in nature. Introducing multiple such dependencies could in principle prevent any escaped mirror bacteria from surviving in the wild. This could be robust against human-caused accidents but not against reckless or deliberate misuse. While less common, a subset of human-caused pathogen exposures are the result of deliberate release. The genetic engineering that introduces these dependencies can be reversed, and as the technology matures, mirror bacteria without any safeguards could be created from scratch.

Treating exposure victims with achiral or racemic antibiotics appears to be feasible. Specifically, fluoroquinolones and sulphonamides should retain potency against mirror bacteria. Further options include synthesizing mirror-image versions of current chiral antibiotics, exploring mirror bacteria biochemistry for novel drug targets and developing conjugate vaccines to prompt the adaptive immune system to recognize mirror bacterial antigens (mRNA vaccines would not work for mirror bacteria).

Whether any of this would translate into effective clinical treatment is less certain. For typical bacterial infections in otherwise healthy patients, antibiotics and vaccines work in concert with, and indeed require, the host immune system to clear infection. In the severely immunocompromised, however, infections can be life-threatening despite potent anti-infective treatment. If mirror bacteria broadly evade immune activation and clearance, every patient would effectively be in that position. Poor control of a treated population of mirror bacteria would be expected to select for antimicrobial resistance, with little further recourse. Setting aside these clinical issues, the logistical challenges are steep: drug development is slow, care for immunocompromised patients is resource-intensive and rapid deployment of new therapeutics against an emerging outbreak has never been easy.

Environmental control, by contrast, looks close to hopeless. Antimicrobials could protect small areas but cannot scale to cover the range of environments mirror bacteria could colonize and would require continuous application to prevent reintroduction. Mirror bacteriophages or engineered predators could serve as biotic controls in bounded settings. In a well-mixed population, a specialist predator cannot by itself drive its prey extinct, since its population would collapse as prey becomes scarce; in finite or spatially structured populations, or where a predator is subsidized by alternative resources, local extinction is achievable. Germline engineering of existing species to resist or outcompete mirror bacteria is conceivable for a few species, but impossible to deploy broadly. Once mirror bacteria enter an environment, they would be essentially impossible to remove – like ink dropped in water.

## Conclusion

While many uncertainties remain about how exactly mirror bacteria would interact with the natural-chirality world, the apparent dangers suggest that direct experimentation is best avoided. The symmetry of chiral mismatch does enable indirect ways to better assess the risk. For example, how well natural-chirality bacteria grow and adapt in an environment of mirror nutrients can provide an analogue of how mirror bacteria would fare in the wild. Expanding the assessment of which bacterial population limiters, including predation, interspecies warfare and immune responses, are stereoselective, and to what degree, would also improve understanding of the nature and scale of the risks. And even if developing effective countermeasures appears unlikely, further research into antibiotic activity against mirror targets could generate novel opportunities.

The concept of mirror life raises deeper insights into life’s origins, history and alternatives. Not only does Earth’s homochirality point to its common origin, but it also raises the question of how alternative biochemistry might have arisen here or elsewhere. There is no convincing evidence for the superiority of l-peptides and d-sugars in nucleic acids, so it is plausible that life also began in the mirror and lost out on Earth at a very early stage but may have prevailed on other planets.

The more beautiful and wonderful side of the mirror biology hypothesis is the implication that everything we know about species interactions – between microbiomes and their animal or plant hosts, within soil and water communities, and between predators and prey – rests on their shared biochemical foundations, developed over eons of evolution. Commensal microbes are tolerated for their ingrained molecular patterns and metabolic processes; invert their chirality, and they become entirely foreign, playing an unprecedented game with their own rules.

## Further Reading

1. Blackmond DG. *The Origin of Biological Homochirality*. Cold Spring Harb Perspect Biol, 2019. p. 11. 10.1101/cshperspect.a032540

2. Xu Y, Zhu TF. Mirror-image T7 transcription of chirally inverted ribosomal and functional RNAs. *Science* 2022;378:405–412. 10.1126/science.abm0646

3. Fan C, Deng Q, Zhu TF. Bioorthogonal information storage in L-DNA with a high-fidelity mirror-image Pfu DNA polymerase. *Nat Biotechnol* 2021;39:1548–1555. 10.1038/s41587-021-00969-6

4. Gaut NJ, Adamala KP. Reconstituting natural cell elements in synthetic cells. *Adv Biol (Weinh*) 2021;5:e2000188. 10.1002/adbi.202000188

5. Kuhn J, Somerville RL. Mutant strains of *Escherichia coli* K12 that use D-amino acids. *Proc Natl Acad Sci USA* 1971;68:2484–2487. 10.1073/pnas.68.10.2484

6. Agashe K, Binder D, Cai Y, Cooper V, et al. Technical report on mirror bacteria: feasibility and risks. *Stanford Digital Repository* 2024. Epub ahead of print 2024. 10.25740/CV716PJ4036

7. Manheim D, Lewis G. High-risk human-caused pathogen exposure events from 1975-2016. *F1000Res* 2022;10:752. 10.12688/f1000research.55114.2

8. Kunjapur AM, Napolitano MG, Hysolli E, Noguera K, Appleton EM, *et al*. Synthetic auxotrophy remains stable after continuous evolution and in coculture with mammalian cells. *Sci Adv* 2021;7:eabf5851. 10.1126/sciadv.abf5851

9. Adamala KP, Agashe D, Belkaid Y, Bittencourt DM de C, Cai Y, *et al*. Confronting risks of mirror life. *Science* 2024;386:1351–1353. 10.1126/science.ads9158

10. Herrmann NC, Stroud JT, Losos JB. The Evolution of “Ecological Release” into the 21st Century. *Trends Ecol Evol* 2021;36:206–215. 10.1016/j.tree.2020.10.019

## Abbreviations

PAMP: pathogen-associated molecular pattern
PRR: pattern recognition receptor

